# Staphylococcus aureus δ-toxin present on skin promotes the development of food allergy in a murine model

**DOI:** 10.3389/fimmu.2023.1173069

**Published:** 2023-05-19

**Authors:** Hiromichi Yamada, Ayako Kaitani, Kumi Izawa, Tomoaki Ando, Anna Kamei, Shino Uchida, Akie Maehara, Mayuki Kojima, Risa Yamamoto, Hexing Wang, Masakazu Nagamine, Keiko Maeda, Koichiro Uchida, Nobuhiro Nakano, Yoshikazu Ohtsuka, Hideoki Ogawa, Ko Okumura, Toshiaki Shimizu, Jiro Kitaura

**Affiliations:** ^1^ Atopy (Allergy) Research Center, Juntendo University Graduate School of Medicine, Tokyo, Japan; ^2^ Department of Pediatrics and Adolescent Medicine, Juntendo University Graduate School of Medicine, Tokyo, Japan; ^3^ Department of Science of Allergy and Inflammation, Juntendo University Graduate School of Medicine, Tokyo, Japan; ^4^ Department of Gastroenterology, Juntendo University Graduate School of Medicine, Tokyo, Japan; ^5^ Department of Immunological Diagnosis, Juntendo University Graduate School of Medicine, Tokyo, Japan; ^6^ Juntendo Advanced Research Institute for Health Science, Juntendo University School of Medicine, Tokyo, Japan

**Keywords:** food allergy, epicutaneous sensitization, murine model, IgE, *Staphylococcus aureus* δ-toxin, IL-1α

## Abstract

**Background:**

Patients with food allergy often suffer from atopic dermatitis, in which *Staphylococcus aureus* colonization is frequently observed. *Staphylococcus aureus* δ-toxin activates mast cells and promotes T helper 2 type skin inflammation in the tape-stripped murine skin. However, the physiological effects of δ-toxin present on the steady-state skin remain unknown. We aimed to investigate whether δ-toxin present on the steady-state skin impacts the development of food allergy.

**Material and methods:**

The non-tape-stripped skins of wild-type, *Kit^W-sh/W-sh^*, or ST2-deficient mice were treated with ovalbumin (OVA) with or without δ-toxin before intragastric administration of OVA. The frequency of diarrhea, numbers of jejunum or skin mast cells, and serum levels of OVA-specific IgE were measured. Conventional dendritic cell 2 (cDC2) in skin and lymph nodes (LN) were analyzed. The cytokine levels in the skin tissues or culture supernatants of δ-toxin-stimulated murine keratinocytes were measured. Anti-IL-1α antibody-pretreated mice were analyzed.

**Results:**

Stimulation with δ-toxin induced the release of IL-1α, but not IL-33, in murine keratinocytes. Epicutaneous treatment with OVA and δ-toxin induced the local production of IL-1α. This treatment induced the translocation of OVA-loaded cDC2 from skin to draining LN and OVA-specific IgE production, independently of mast cells and ST2. This resulted in OVA-administered food allergic responses. In these models, pretreatment with anti-IL-1α antibody inhibited the cDC2 activation and OVA-specific IgE production, thereby dampening food allergic responses.

**Conclusion:**

Even without tape stripping, δ-toxin present on skin enhances epicutaneous sensitization to food allergen in an IL-1α-dependent manner, thereby promoting the development of food allergy.

## Introduction

The prevalence of food allergy is increasing, particularly in western countries, thereby poising a critical public health problem. Generally, the sensitization to food allergen via several routes, accompanied by the production of food allergen-specific immunoglobulin E (IgE) antibody (Ab), plays an important role in the development of food allergy, which is caused by the oral intake of the same allergen. The occurrence of food allergy is characterized by diarrhea and anaphylaxis in severe cases. These symptoms are caused by chemical mediators mainly released from mast cells, which are activated by the engagement of high affinity IgE receptor (FcεRI) on their surfaces with food allergen and its specific IgE (1–6). Recent advances highlight that epicutaneous sensitization to food allergen is important in developing food allergy (7–11). Food allergy is associated with atopic dermatitis, in which skin barrier dysfunctions are critical pathogenic factors (1–3, 12, 13). Further, the skin of patients with atopic dermatitis is frequently colonized by *Staphylococcus aureus* (*S. aureus*), which is known to produce several exotoxins. Among them, δ-toxin (also called phenol-soluble modulin (PSM)-γ), which belongs to the peptide toxin family of PSM, directly activates mast cells and promotes T helper 2 (Th2) type skin inflammation with increased IgE production (12–14). The specific receptor of δ-toxin and its function in immune cells are not completely understood (12). PSMα peptides (PSMα1-4) are highly cytotoxic to a variety of cells, while other PSM, including δ-toxin, exhibits limited cytotoxic activity (12–14). In murine model of epicutaneous infection of S. aureus infection, PSMα induces the release of interleukin (IL)-1α and IL-36α that orchestrate IL-17-dependent skin inflammation (15, 16). Furthermore, IL-36α also directly stimulates B cells to enhance IgE production (17). In the skins of patients with atopic dermatitis, expression of IL-1α, IL-1β, IL-18, IL-33, and IL-36α, which belong to IL-1 family, is known to be upregulated in keratinocytes (18–21). However, the context-dependent functions of these cytokines in epicutaneous sensitization is not yet clear.

Mouse models have been used to study food allergy induced by oral challenges with ovalbumin (OVA) following intraperitoneal sensitization with OVA plus alum adjuvant. Mice with food allergy exhibited high serum levels of OVA-specific IgE and mast cell protease 1 (MCPT1), which is a mucosal mast cell activation marker, and intestinal mast cell hyperplasia. In these models, mast cells and OVA-specific IgE are indispensable for the induction of food allergy (4–6). Alternatively, several models of food allergy in mice after epicutaneous sensitization have been developed recently (8–11). Epicutaneous treatment with OVA on the tape-stripped skins of mice induces Th2/T follicular helper (Tfh) responses through several mechanisms. These mechanisms involve epithelial cell-derived cytokines such as thymic stromal lymphopoietin (TSLP) and IL-33 and skin immune cells such as conventional dendritic cell 2 (cDC2). Among several antigen-presenting cells, cDC2 is justified as the most prominent in inducing Th2 responses. Under inflammatory conditions, cDC2 uptakes food allergen in the dermis and moves to the draining lymph node (LN), where it presents the food antigens to naïve CD4^+^ T cells to induce Th2/Tfh responses (22–26). Recent reports have shown that tape-stripping alone causes the release of IL-33 in skin, which induces the expansion of intestinal mast cells via keratinocyte-derived IL-33-intestinal type 2 innate lymphoid cell (ILC2) axis and enhances IgE-mediated food allergic responses (7, 8).

The present study aimed to investigate whether δ-toxin of *S. aureus* is implicated in the development of food allergy following epicutaneous sensitization of steady-state skin. To remove the effects of tape stripping on immune responses and to clarify the exact roles of δ-toxin on skin with normal barrier function, we used murine model of food allergy after epicutaneous treatment of the non-tape-stripped skin.

## Materials and methods

### Mice

We used wild-type (WT) BALB/c mice (Japan SLC, Hamamatsu, Japan) and *Kit^W-sh/W-sh^* and ST2-deficient mice on the BALB/c background were used (6, 27–29). All animal experiments were approved by the ethical committee of Juntendo University (approval numbers 310050 and 310051).

### Antibodies and other reagents

The following antibodies (Abs) were used: Fluorescein isothiocyanate (FITC)-anti- FcεRIα (MAR-1) (eBioscience), MHC Class II (M5/114.15.2) (BioLegend), phycoerythrin (PE)-anti-CD24 (M1/69) and CD103 (2E7) (BioLegend), PE-cyanin 7 (Cy7)-anti-CD64 (X54-5/7.1) and EpCAM (G8.8) (BioLegend), Allophycocyanin (APC)-c-Kit (2B8) (eBioscience), APC-Cy7-CD45 (30-F11) and Zombie (B279801) (BioLegend), Peridinin Chlorophyll Protein Complex (PerCP)-Cy5.5-anti-CD45 (30-F11) and CD11b (M1/70) (BioLegend), Streptavidinanti (BioLegend), Brilliant Violet (BV) 421-anti-CD11c (N418), and CD11b (M1/70) (BioLegend), Biotin anti-CD11c (N418), CD19 (1D3), CD3 (145-2C11), and CD11b (M1/70) (Tonbo Biosciences), Biotin anti-F4/80 (RME-1), FcεRIα (MAR-1), CD49b (DX5), and Ly-6G/Ly-6C (Gr-1) (BioLegend). Cytokines were purchased from R&D Systems. OVA (Grade V) was purchased from Sigma. Alexa Fluor 647-conjugated OVA (OVA-AF647) was generated by labeling OVA with Alexa Fluor™ 647-NHS Ester (Thermo Fisher Scientific) following manufacturer’s instructions. The toxins δ-toxin (MAQDIISTIGDLVKWIIDTVNKFTKK) and PSMα3 (MEFVAKLFKF FKDLLGKFLG NN) were synthesized (GL Biochem, Shanghai, China).

### Cells

Axillary LNs were isolated from the mice and single-cell suspensions of these LNs were prepared. Bone marrow-derived mast cells (BMMCs) were generated as previously described (6, 27). Briefly, murine BM cells were incubated in RPMI 1640 medium including 10% FCS in the presence of 10 ng/mL IL-3. Five weeks after incubation, more than 95% pure population of FcεRI^+^c-kit^+^ mast cells (BMMCs) were generated. Alternatively, murine BM cells were incubated in RPMI 1640 medium including 10% FCS in the presence of 20 ng/mL GM-CSF for 10 days to generate CD11b^+^CD11c^+^ BM-derived dendritic cells (BMDCs). Murine keratinocytes were isolated as described previously (30, 31). To separate the epidermis from the dermis, skins of newborn mice were treated with 5 mg/mL DISPASE II (FUJIFILM) overnight at 4 °C. The mechanically-separated epidermis was washed with phosphate-buffered saline (PBS) and incubated with CnT-ACCUTASE-100 (CELLNTEC) for 20 min at room temperature to obtain the keratinocytes. Murine keratinocytes were cultured in CnT-Prime, Epitherial Culture Medium (CELLNTEC) using 1.2 mM CaCl_2_ on collagen-coated plates to induce keratinocyte differentiation.

### Mouse model of food allergy following epicutaneous sensitization

Epicutaneous sensitization was performed as previously described, with a few modifications (7, 10, 11, 13). Female mice aged 8-10 weeks were anesthetized, and depilatory cream was applied on their abdominal skins. Thereafter, OVA (200 μg in 100 μL saline) and δ-toxin (200 μg in 100 μL saline) or 100 μL saline were applied once a week for six weeks (on days 0, 7, 14, 21, 28, and 35) to the abdominal skins of the mice that had not been subjected to tape stripping. One week after the final epicutaneous treatment, the mice were intragastrically gavaged with OVA (50 mg in 200 μL saline) every 2 d for a total seven to twelve times. Diarrhea was assessed by visually monitoring mice for up to 30 minutes after intragastric challenge of OVA. Mice excreting loose or liquid stools were recorded as mice with diarrhea. The frequency of diarrhea (%) means the percentage of the mice with diarrhea among all the mice tested (6).

### ELISA measurements for cytokines, MCPT-1, IgE, and OVA-specific IgE

ELISA kits for IL-4, IL-13, IL-33, IL-1α, IL-1β, IL-25, and TSLP (R&D Systems), mast cell protease-1 (MCPT-1) (eBioscience), and high mobility group box 1 (HMGB1) (Promega) were used to measure their concentrations in serum, culture supernatants, and skin tissue homogenates. ATP levels in culture supernatants and skin tissue homogenates were measured using the CellTiter-Glo Assay (Promega) About 10 mg skin samples were minced using scissors and placed in 200 μL of PBS that contains protease inhibitor cocktail (FUJIFILM) at 4°C for 30 min. Thereafter, it was centrifuged at 12000 g at 4°C for 30 min, and the supernatants were used for cytokine detection. The concentrations of OVA-specific IgE and IgG1 were determined with luminescence ELISA by using OVA, anti-IgE Ab (R35-118) (BD Pharmingen), anti-IgG1 Ab (1070–08) (Southern Biotech), streptavidin-horseradish peroxidase (HRP), and TMB substrate solution (BD Biosciences), as previously described (6, 29).

### Histology

Sections of mice jejunum were obtained approximately 10 cm from the pyloric sphincter and fixed in 10% formalin and embedded in paraffin. These paraffin-embedded sections of the jejunum and skin were stained with chloroacetate esterase for the quantification of mast cells, as previously described (6, 27, 29). Alternatively, paraffin sections of the skin were stained with Hematoxylin and Eosin (27).

### *Ex vivo* Th2 responses

Single-cell suspensions of axillary LN cells (2 × 10^6^) were cultured in the presence of 25 μg/mL OVA for 4 d to measure cytokines (IL-4 and IL-13) in the supernatants of the cultures suspensions (6, 29).

### Real-time PCR

RNA extraction, cDNA synthesis, and real-time PCR were performed as previously described (27). The jejunum tissues were homogenized using the tissueLyser (Qiagen), and total RNA was extracted using RNeasy Lipid Tissue Mini Kit (Qiagen) according to the manufacturer’s instructions. cDNA was synthesized from total RNA using the ReverTra Ace qPCR RT kit (Toyobo). Real-time PCR was performed with the Step One Plus Real-Time PCR System (Thermo Fisher Scientific) using the SYBR Green PCR Master Mix (Applied Biosystems, Life technologies). The primers used were shown (**Supplementary Table 1**). The mRNA expression levels were quantified with the comparative method using StepOne Software. The housekeeping gene 18S rRNA was used for normalization.

### Flow cytometry

Flow cytometric analysis was performed with FACSVerse (BD Biosciences), as previously described (6, 27–29), and the obtained data were analyzed using FlowJo software (Tree Star). cDC2 were identified as CD45^+^CD11c^+^ MHC class II^+^ CD11b^+^CD103^-^EpCAM^-^, as suggested by some recent studies (22, 32–36).

### Measurements of the percentage of OVA-AF647-positive cells among cDC2 in the skin or axillary LN or among MHC Class II^high^ BMDCs

To measure the percentages of OVA-AF647-positive cells *in vivo*, depilatory cream was applied on the abdominal skins of the mice. Thereafter, OVA-AF647 (200 μg in 100 μL saline) and δ-toxin (200 μg in 100 μL saline) or 100 μL saline were applied on days 0 and 7 to the abdominal skins of the mice that had not been subjected to tape stripping. Twenty-four hours after the last epicutaneous treatment, the percentage of OVA-AF647 among skin cDC2 or axillary LN cDC2 obtained from the mice were measured using flow cytometry, as previously described (32). BMDCs were cultured in the presence of 0 or 10 ng/mL IL-1α for 12 h, and then were incubated with 0, 100, or 300 ng/mL OVA-AF647 for 1 h to measure the percentages of OVA-AF647 among CD11b^+^CD11c^+^MHC Class II^high^ BMDCs using flow cytometry.

### Evaluation of cytotoxicity

The number of the non-viable cells were estimated by CytoTox-ONE™ Homogeneous Membrane Integrity Assay (Promega, Madison, WI), which is a lactate dehydrogenase (LDH) release-based assay that uses culture supernatants.

### Statistical analyses

Results are expressed as means ± standard deviation (SD). Ordinary one-way analysis of variance (ANOVA) with Tukey’s multiple comparisons was used in **Figures 1**–**5**, and **Supplementary Figure 1**. Welch’s *t*-test was used in **Figures 6**, **7**; **Supplementary Figures 2**-**4**. Differences were compared between groups, and *p < 0.05 or **p < 0.01 was considered statistically significant.

**Figure 1:**
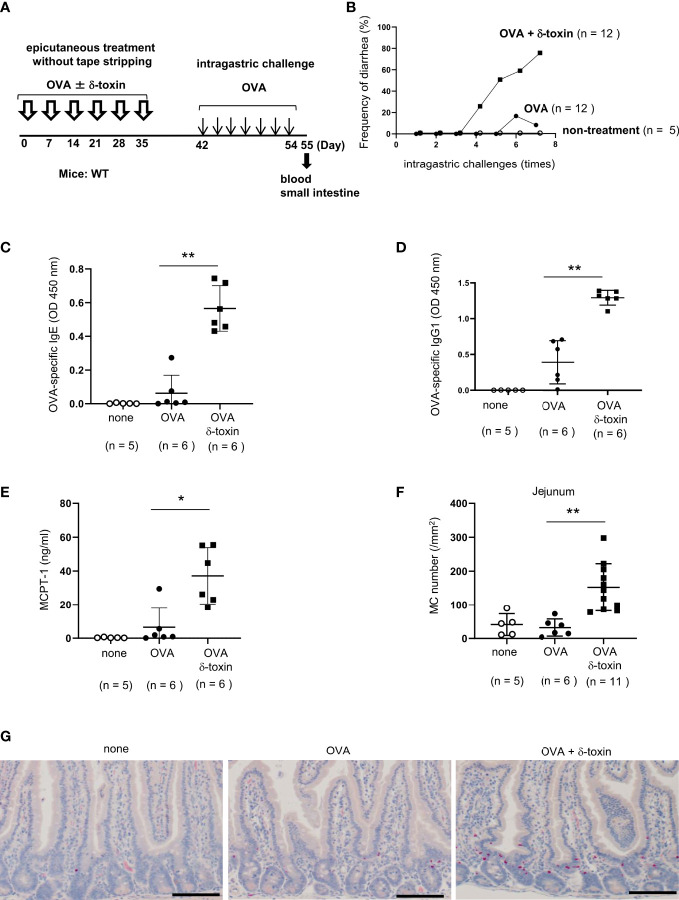
δ-toxin present on the non-tape-stripped skin strongly induced food allergic responses following epicutaneous sensitization to food allergens in a murine model. **(A)** Experimental design to investigate the occurrence of for food allergy after intragastric administration of OVA in WT mice that had been epicutaneously treated or not with OVA ± δ-toxin once a week for six weeks. Blood samples were taken, and small intestines were isolated on day 56. **(B)** Frequency of diarrhea in OVA-challenged mice after epicutaneous treatment with OVA ± δ-toxin on the non-tape-stripped skin or after non-treatment. **(C–E)** Serum levels of **(C)** OVA-specific IgE, **(D)** OVA-specific IgG1, and **(E)** MCPT-1 in the mice after the final administration of OVA. **(F)** The numbers of jejunum mast cells of the mice after the final administration of OVA. **(G)** Jejunum sections stained with chloroacetate esterase (scale bars, 100 μm). Mast cells stain red. **(B, F)** Data are pooled from two independent experiments. **(C–E)** Data are representative of two independent experiments. Means ± SD have been plotted. *, *P* < 0.05, **, *P* < 0.01.

**Figure 2:**
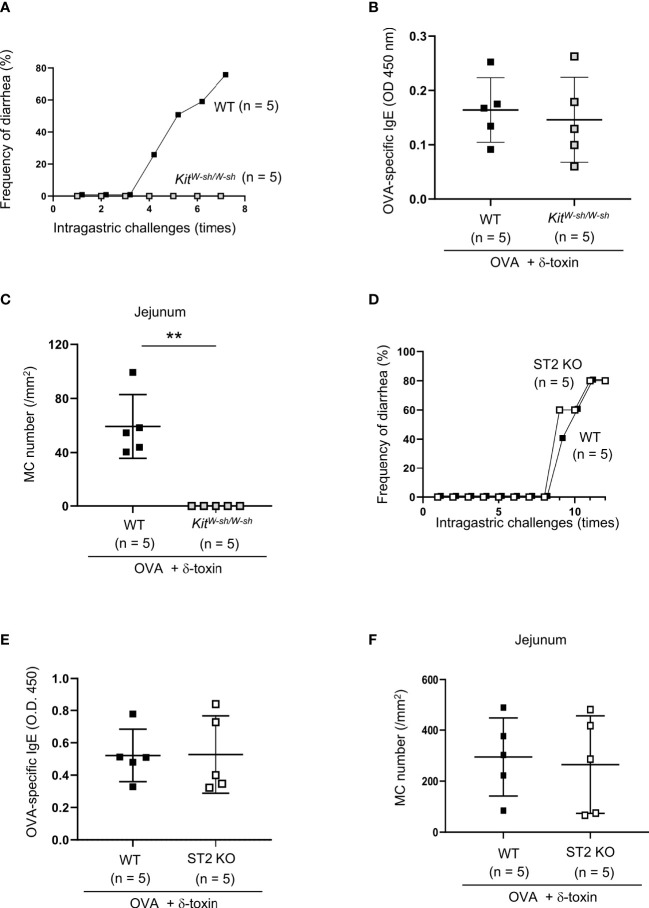
IL-33-ST2 signaling is dispensable for OVA-challenged food allergic responses after epicutaneous treatment with OVA and δ-toxin on the non-tape-stripped skin. **(A, D)** Frequency of diarrhea in OVA-administered mice after epicutaneous treatment with OVA ± δ-toxin on the non-tape-stripped skin of **(A)** WT and *Kit^W-sh/W-sh^* mice and **(D)** WT and ST2 knockout (KO) mice. **(B, E)** Serum levels of OVA-specific IgE in **(B)** WT and *Kit^W-sh/W-sh^* mice and **(E)** WT and ST2 KO mice after the final administration of OVA. **(C, F)** The numbers of jejunum mast cells in **(C)** WT and *Kit^W-sh/W-sh^* mice and **(F)** WT and ST2 KO mice after the final administration of OVA. **(A–F)** Data are representative of two independent experiments. **(B, C, E, F)** Means ± SD have been plotted. **, *P* < 0.01.

**Figure 3:**
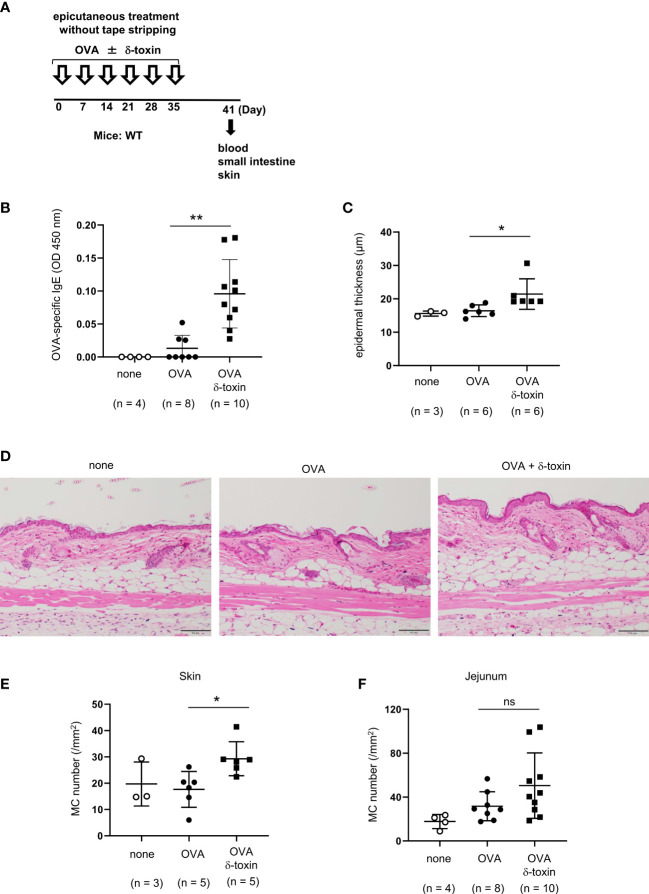
δ-toxin present on the non-tape-stripped skin enhanced epicutaneous sensitization to food allergen in a murine model. **(A)** Experimental design for epicutaneous sensitization. WT mice were epicutaneously treated or not with OVA ± δ-toxin once a week for six weeks on the non-tape-stripped abdominal skin. On day 42, blood samples were obtained, and skins and small intestines were isolated. **(B)** Serum levels of OVA-specific IgE. **(C)** The thickness of epidermis. **(D)** Skin sections stained with hematoxylin and eosin (scale bars, 100 μm). **(E, F)** The numbers of mast cells in the **(E)** skin and **(F)** jejunum. **(B, F)** Data are pooled from two independent experiments. **(C, E)** Data are representative of two independent experiments. Means ± SD have been plotted. *, *P* < 0.05, **, *P* < 0.01. ns, not significant.

**Figure 4:**
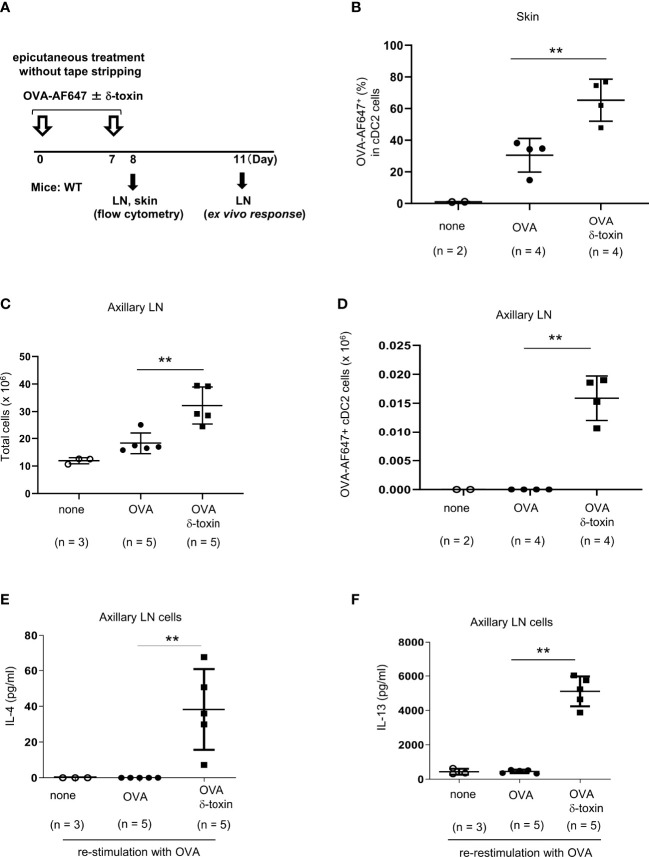
δ-toxin present on the non-tape-stripped skin strongly induced the translocation of OVA-loaded cDC2 from skin to draining LN in murine model. **(A)** Experimental design for analyzing dendritic cells in skin and axillary LNs. WT mice were epicutaneously treated or not with OVA-AF647 ± δ-toxin on the non-tape-stripped abdominal skin on days 0 and 7. Samples of skin were isolated on Day 8 and axillary LNs were isolated on Day 8 or 11. **(B)** The percentage of OVA-AF647-positive cells among skin cDC2 from the mice 24 h after the final treatment. **(C, D)**, **(C)** Total cells and **(D)** AF-647-positive cDC2 in axillary LN of mice 24 h after the final treatment. **(E, F)** Axillary LN cells purified from the mice 96 h after the final treatment were re-stimulated with 25 μg/mL OVA for 4 days. Concentrations of **(E)** IL-4 and **(F)** IL-13 in the culture supernatants of axillary LN cells. **(B–F)** Data are representative of two independent experiments. Means ± SD have been plotted. **P < 0.01.

**Figure 5:**
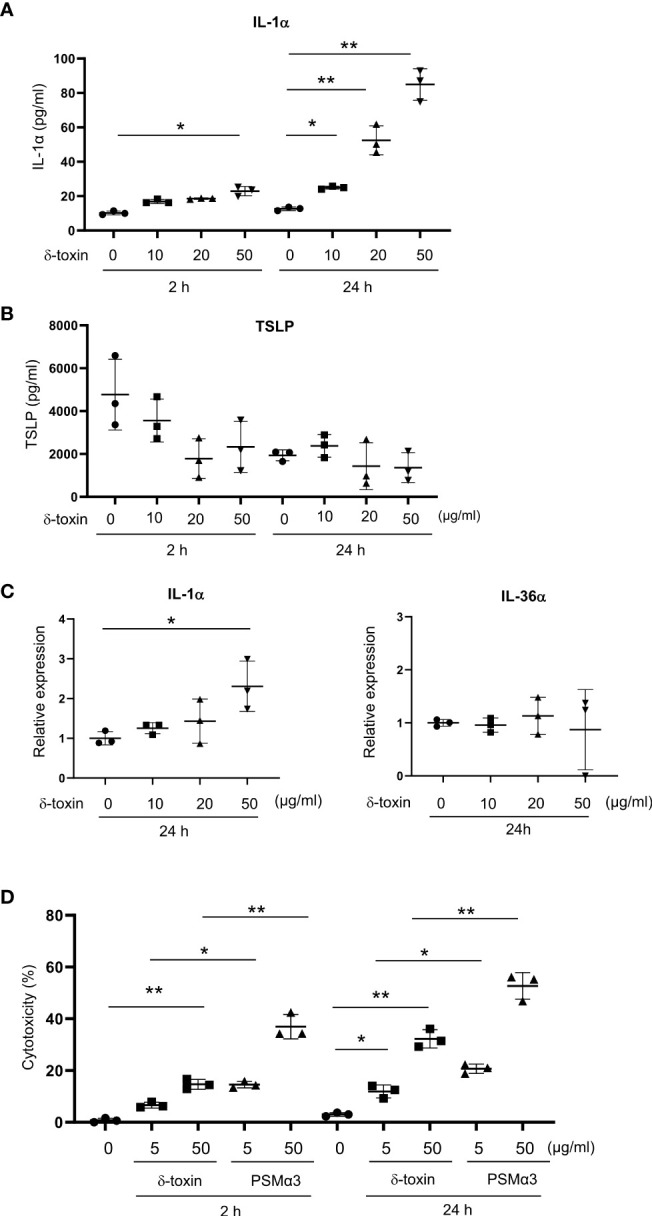
Murine keratinocytes released IL-1α in response to stimulation with δ-toxin. Murine keratinocytes were stimulated with different concentrations of δ-toxin for 2 or 24 h, as indicated. **(A, B)** Concentrations of **(A)** IL-1α and **(B)** TSLP in the culture supernatants. **(C)** Relative expression levels of mRNA of IL-1α and IL-36α in the δ-toxin-stimulated keratinocytes. **(D)** The percentage of dead cells. **(A–D)** Data are representative of three independent experiments. Means ± SD have been plotted. *P < 0.05 or **P < 0.01.

**Figure 6:**
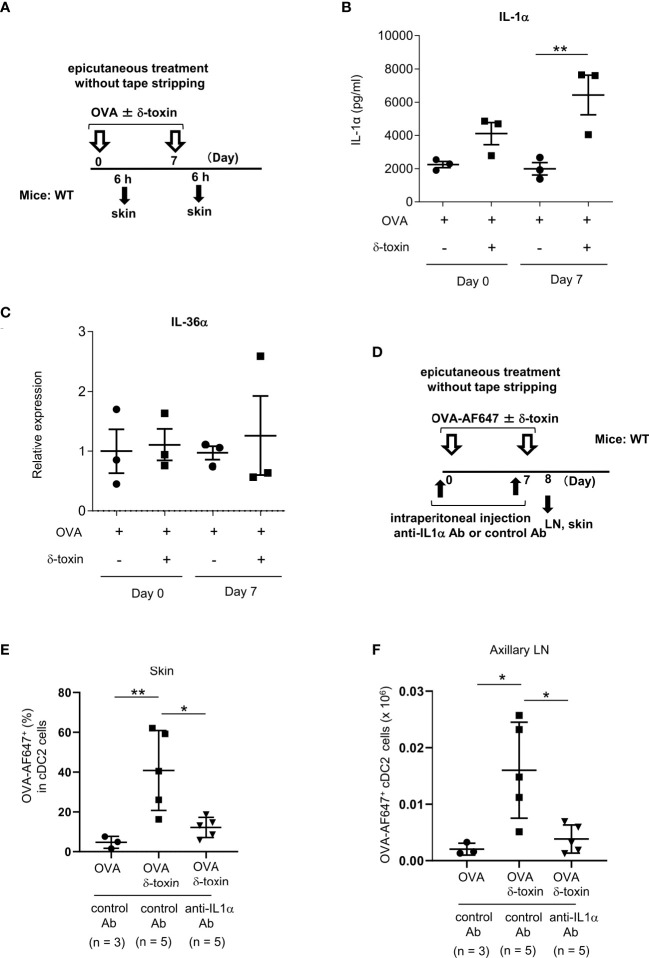
Pretreatment with anti-IL-1α Ab decreased the δ-toxin-mediated translocation of OVA-loaded cDC2 from skin to draining LN in murine model. **(A)** Experimental design for analyzing the cytokine levels in skin tissues. **(B, C)** Protein levels of IL-1α in skin tissue homogenates **(B)** and mRNA levels of IL-36α in skin tissues **(C)** obtained from the mice 6 h after the first or second epicutaneous treatment with OVA ± δ-toxin on the non-tape-stripped abdominal skin. **(D)** Experimental design for analyzing dendritic cells in skin and axillary LN. Non-tape-stripped abdominal skin of WT mice were epicutaneously treated with OVA-AF647 ± δ-toxin on days 0 and 7. The effects of intraperitoneal administration of anti-IL-1α Ab or control Ab were examined. Skins and axillary LNs were isolated on day 8. **(E)** The percentage of OVA-AF647-positive cells among skin cDC2 from the mice 24 h after the last treatment. **(F)** AF-647-positive cDC2 in axillary LN from the mice 24 h after the last treatment. **(B, C, E, F)** Data are representative of two independent experiments. Means ± SD have been plotted. *, *P* < 0.05, **, *P* < 0.01.

**Figure 7:**
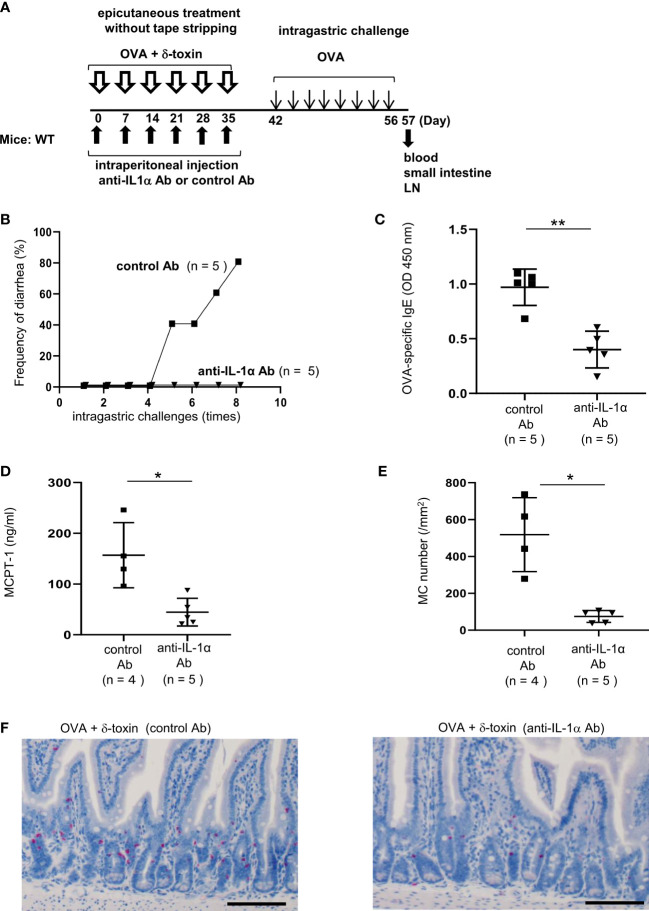
Pretreatment with anti-IL-1α Ab dampened δ-toxin-mediated, OVA-induced food allergic responses in murine model. **(A)** Experimental design for food allergy of intragastrically OVA-administered WT mice that had been epicutaneously treated with OVA + δ-toxin once a week for six weeks on the non-tape-stripped abdominal skin. The effects of intraperitoneal administration of anti-IL-1α Ab or control Ab were examined. Blood samples were taken, and small intestines were isolated on day 57 **(B)** Frequency of diarrhea in OVA-administered mice. **(C–E)** The serum levels of **(C)** OVA-specific IgE and **(D)** MCPT-1 in the mice after the last challenge with OVA. **(E)** The numbers of jejunum mast cells of the mice after the last challenge with OVA. **(F)** Jejunum sections stained with chloroacetate esterase (scale bars, 100 μm). Mast cells stain red. **(B-F)** Data are representative of two independent experiments. Means ± SD have been plotted. *P < 0.05 or **P < 0.01.

## Results

### δ-toxin present on the non-tape-stripped skin strongly induced food allergic responses following epicutaneous sensitization to food allergens in a murine model

To investigate whether an epicutaneous treatment with *S. aureus* δ-toxin on steady-state skin contributes to the development of food allergy, we used a murine model of OVA-induced food allergy. We avoided any impact of tape stripping on the immune cells of skin and small intestine. For this, Balb/c mice that had not been subjected to tape stripping were selected, and OVA was applied to their abdominal skin either with or without δ-toxin once a week for six weeks. Between days 42 and 55, these mice were intragastrically administered OVA every 2 days for a total of seven times (**Figure 1A**). We found that the epicutaneous treatment with OVA in the presence of δ-toxin induced more frequent diarrhea after OVA administration than that with OVA alone did (**Figure 1B**). In contrast, OVA administration without prior epicutaneous treatment did not induce diarrhea at all (**Figure 1B**). We also measured the serum levels of OVA-specific IgE and IgG1 as well as MCPT-1 in the mice after the final intragastric administration of OVA. The results revealed that epicutaneous treatment with OVA together with δ-toxin significantly increased all levels compared to those observed following treatment with OVA alone (**Figures 1C–E**). Histological examination revealed that the numbers of jejunum mast cells were higher in the mice subjected to epicutaneous treatment with OVA and δ-toxin than in those treated with OVA alone (**Figures 1F, G**). In addition, real time-PCR analysis showed that mRNA levels of the Th2 cytokine IL-4 and MCPT-1 in mouse jejunum tissues were higher in the mice epicutaneously treated with OVA and δ-toxin (**Supplementary Figures 1A–D**). Thus, δ-toxin present on the skin strongly induced food allergic responses with the Th2 skewing following epicutaneous treatment with OVA even without the procedure of tape stripping (12, 13).

### IL-33-ST2 signaling is dispensable for OVA-challenged food allergic responses following epicutaneous treatment with OVA plus δ-toxin on the non-tape-stripped skin

To test whether the OVA-challenged food allergic responses following epicutaneous treatment with OVA plus δ-toxin on the non-tape-stripped skin depend on mast cells, we used the same model in WT and mast cell-deficient *Kit^W-sh/W-sh^* mice. The results showed that intragastric challenges with OVA caused frequent diarrhea in WT mice, but not in *Kit^W-sh/W-sh^* mice, which had no detectable mast cells in the jejunum tissues (**Figures 2A, C**). However, the serum levels of OVA-specific IgE after final challenges with OVA were comparable between both mice (**Figure 2B**). Hence, intestinal mast cells are indispensable for OVA-challenged food allergic responses, but mast cells are not necessary for OVA-specific IgE production in this model.

As IL-33 plays an important role in food allergic responses following epicutaneous treatment with OVA on the tape-stripped skin, we also investigated whether ST2, a receptor for IL-33, is involved in δ-toxin-mediated food allergy in our model. We performed the same treatment in WT and ST2-deficient mice, and found that ST2 deficiency failed to influence the frequency of diarrhea after OVA administration (**Figure 2D**). In addition, no difference was observed in the serum levels of OVA-specific IgE and the numbers of jejunum mast cells between WT and ST2-deficient mice following the OVA challenges (**Figures 2E, F**). Hence, IL-33/ST2 signaling is not essential for OVA-challenged food allergic responses following epicutaneous treatment with OVA plus δ-toxin on the non-tape-stripped skin.

### δ-toxin present on the non-tape-stripped skin enhanced epicutaneous sensitization to food allergens in a murine model

We clarified the role of δ-toxin present on the non-tape-stripped skin by analyzing WT mice on day 41 before intragastric administration. Notably, δ-toxin remarkably increased the serum levels of OVA-specific IgE after the last epicutaneous OVA treatment even without tape-stripping (**Figures 3A, B**). We also confirmed that mast cell deficiency did not influence δ-toxin-mediated production of OVA-specific IgE in this model (**Supplementary Figure 2**). In addition, as revealed by histological examination, the mice treated epicutaneously with OVA and δ-toxin exhibited a slight increase in epidermal thickness and mast cell numbers in the skin compared to those that were treated with OVA alone (**Figures 3C–E**). In contrast, δ-toxin present on the non-tape-stripped skin did not significantly increase jejunum mast cell numbers (**Figure 3F**). Hence, *S. aureus* δ-toxin present on the non-tape-stripped skin strongly induced epicutaneous sensitization to food allergens independently of mast cells, thereby resulting in the food allergic responses after intragastric challenges with the same allergen in this model.

### δ-toxin present on the non-tape-stripped skin strongly induced the translocation of OVA-loaded cDC2 from skin to the draining LN in a murine model

We assessed whether δ-toxin influences the uptake of OVA by skin cDC2 and/or the translocation of OVA-loaded cDC2 to the draining LN. We applied OVA-AF647 with or without δ-toxin on the non-tape-stripped skin on days 0 and 7. About 24 h after the final epicutaneous treatment, we measured the percentages of AF647-positive cells among skin cDC2 and AF647-positive cDC2 numbers in axillary LN (**Figure 4A**). The percentages of AF647-positive cells among skin cDC2 were higher in those mice that were epicutaneusly treated with AF647-OVA and δ-toxin than in those treated with AF647-OVA alone, although the percentages of skin cDC2 among total skin cells were lower in the former mice than in the latter mice (**Figure 4B**; **Supplementary Figure 3A**). In addition, we found a significant increase in cDC2 numbers and AF647-positive cDC2 numbers as well as total cell numbers in axillary LN (**Figures 4C, D**; **Supplementary Figure 3B**). It should be noted that the deficiency of mast cells or ST2 did not influence δ-toxin-mediated increase of AF647-positive cDC2 numbers in axillary LN in the same model (**Supplementary Figures 3C–E**). Moreover, the concentrations of IL-4 and IL-13 were higher in the supernatants of OVA-restimulated axillary LN cells from the mice that were epicutaneously treated with OVA and δ-toxin compared to those in mice treated with OVA alone (**Figures 4E, F**). These results indicated that even without tape-stripping, epicutaneously treated δ-toxin enhanced the uptake of OVA from cDC2 in the skin, and enhanced the translocation of OVA-loaded cDC2 from skin to the draining LN, which resulted in enhanced sensitization to OVA.

To examine whether δ-toxin on skin plays a prominent role in the development of food allergy among the peptide toxin family of PSM, we compared the difference in the effects of PSMα3, which is a highly cytotoxic peptide, and δ-toxin in the same model. Analysis of OVA-loaded cDC2 in axillary LN showed that AF647-positive cDC2 numbers were significantly lower in PSMα3-treated mice than in δ-toxin-treated mice (**Supplementary Figures 4A, B**). Moreover, the serum levels of OVA-specific IgE were also significantly lower in PSMα3-treated mice on day 41 before OVA challenges (**Supplementary Figures 4C, D**). Consistently, PSMα3-treated mice exhibited less frequent diarrhea as compared with δ-toxin-treated mice (**Supplementary Figure 4E**). The number of jejunum mast cells after the last OVA administration tended to be lower in PSMα3-treated mice compared to that in δ-toxin-treated mice (**Supplementary Figure 4F**). Thus, δ-toxin present on the non-tape-stripped skin induced OVA-specific IgE production more strongly than PSMα3. This can likely be due to the enhanced translocation of OVA-loaded cDC2 from skin to the draining LN in this model.

### Murine keratinocytes released IL-1α in response to stimulation with δ-toxin

As the major target cells of δ-toxin on the non-tape-stripped skin were likely keratinocytes in the epidermis, we stimulated the murine primary keratinocytes with different concentrations of δ-toxin for 2 h or 24 h. Notably, the concentrations of IL-1α in the culture supernatants increased with an increase in incubation time and δ-toxin concentration (**Figure 5A**). We found that keratinocytes constitutively released TSLP, whose concentrations did not increase after δ-toxin stimulation (**Figure 5B**). We could not detect the protein levels of IL-1β, IL-18, IL-25, or IL-33 in the culture supernatants of δ-toxin-stimulated keratinocytes. Real time PCR analysis showed that stimulation with δ-toxin slightly increased the mRNA levels of IL-1α, but it did not of alter those of IL-36α in murine keratinocytes (**Figure 5C**). However, mRNA levels of putative receptors for δ-toxin, including several formyl peptide receptors, were low in keratinocytes (**Supplementary Figure 5A**). Instead, stimulation with δ-toxin induced cell death of keratinocytes in a time- and concentration-dependent manner. However, PSMα3 showed higher cytotoxic effect on keratinocytes than δ-toxin (**Figure 5D**). In accordance with this, stimulation with PSMα3 more strongly induced the release of damage-associated molecular patterns (DAMPs) such as IL-1α, ATP, and HMGB1 than that with δ-toxin (**Supplementary Figures 5B–D**). In addition, stimulation with IL-1α increased the mRNA levels of IL-1α in murine keratinocytes (**Supplementary Figure 5E**). Accordingly, it is possible to speculate that δ-toxin induced the release of IL-1α from keratinocytes through passive cell death, which in turn transcriptionally upregulated IL-1α in an autocrine manner.

### Pretreatment with anti-IL-1α Ab decreased the δ-toxin-mediated translocation of OVA-loaded cDC2 from skin to the draining LN in a murine model

We measured the protein levels of IL-1α in skin tissues from the mice epicutaneously treated with OVA ± δ-toxin for the indicated periods (**Figure 6A**). The results showed that IL-1α levels in skin tissues were higher in the mice treated with OVA plus δ-toxin than those in mice treated with OVA alone, six hours after the second epicutaneous treatment. This suggests that the presence of δ-toxin caused IL-1α production in the local skin even when the skin was not stripped using tape (**Figure 6B**). However, real time PCR analysis showed that mRNA levels of IL-36α were not up-regulated in δ-toxin-treated skin in the same model (**Figure 6C**). Notably, IL-1α levels in skin tissues were higher in the mice epicutaneously treated with OVA plus δ-toxin than in those with OVA plus PSMα3, while there was no significant difference in levels of ATP and HMGB1 between the two groups in the same model (**Supplementary Figure 6**). We found that pretreatment with a blocking Ab against IL-1α, but not with a control Ab, substantially reduced the percentages of AF647-positive cells among skin cDC2 and AF647-positive cDC2 numbers in axillary LN in the mice epicutaneously treated with OVA-AF647 plus δ-toxin (**Figures 6D–F**). In addition, stimulation with IL-1α increased the uptake of OVA-AF647 in MHC Class II^high^ BMDCs (**Supplementary Figure 7**). Overall, these results suggested that the δ-toxin-mediated release of IL-1α contributes to the uptake of OVA from skin cDC2 and the translocation of OVA-loaded cDC2 from skin to the draining LN, leading to an efficient sensitization to OVA in this model.

### Pretreatment with anti-IL-1α Ab dampened δ-toxin-mediated, OVA-induced food allergic responses in a murine model

To investigate the role of IL-1α in food allergies mediated by δ-toxin present on the steady-state skin, we pretreated the mice with anti-IL-1α Ab or control Ab (**Figure 7A**). Notably, pretreatment with anti-IL-1α Ab, but not with control Ab, suppressed δ-toxin-mediated, OVA administration-induced diarrhea in our model (**Figure 7B**). Consistently, pretreatment with anti-IL-1α Ab strongly reduced the serum levels of OVA-specific IgE and MCPT-1 and the numbers of jejunum mast cells (**Figures 7C–F**) as well as the epidermal thickness (**Supplementary Figure 8**) in the OVA-administered mice following epicutaneous treatment with OVA plus δ-toxin. Thus, pretreatment with anti-IL-1α Ab suppressed the skin inflammation, the sensitization to food allergen, and subsequent food allergic responses in this model. We concluded that the presence of δ-toxin on the non-tape-stripped skin induced the release of IL-1α from keratinocytes, which promoted the uptake of food allergens by cDC2 in the skin and the subsequent migration of OVA-loaded cDC2 to the draining LN. This, in turn, leads to the efficient sensitization to food allergens in the development of food allergy.

## Discussion

To elucidate the mechanisms underlying food allergy following epicutaneous sensitization, tape stripping prior to epicutaneous treatment with food allergen has been widely used in murine models (8–11). Tape stripping mimics mechanical skin injury caused by scratching in patients with atopic dermatitis. However, recent studies have demonstrated that tape stripping alone causes skin epithelial damage, resulting in the local release of IL-33, which stimulates intestinal mast cell hyperplasia via intestinal ILC2 (7). Moreover, tape stripping-derived IL-33 also enhances IgE-dependent food allergic responses via mast cells (8). In addition, when tape-stripped skins of mice were treated with δ-toxin or exposed to δ-toxin-producing *S. aureus*, δ-toxin induced mast cell-dependent Th2 skin inflammation with increased IgE production (12, 13). In models using tape stripping, δ-toxin may directly stimulate the degranulation of mast cells in the dermis. However, the presence of δ-toxin-producing *S. aureus* in humans does not always translate into atopic dermatitis with skin barrier disruption. In most cases, δ-toxin may be present on the normal skin with intact barrier function. In the present study, we aimed to investigate whether δ-toxin present on the steady-state skin contributes to the development of food allergy following epicutaneous sensitization. We used a murine model in which skin without tape stripping was treated with OVA in the presence or absence of δ-toxin prior to intragastrical administration of OVA. It seems therefore that this model is not always a representative of food allergy in patients with atopic dermatitis, but recapitulates food allergy in δ-toxin-producing *S. aureus*-colonized individuals who have suffered from mild atopic dermatitis or have not yet developed atopic dermatitis. Notably, repeated epicutaneous treatment with OVA and δ-toxin on the non-tape-stripped skin induced a mild epidermal thickness and OVA-specific IgE production, leading to the increase in the frequency of diarrhea and number of jejunum mast cells after intragastric OVA challenges. This was not observed in treatments with OVA alone. In addition, analysis of mast cell- and ST2-deficient mice revealed that both mast cells and ST2 are dispensable for OVA-specific IgE production in mice; however, intestinal mast cells, but not ST2, are indispensable for food allergic responses induced after OVA administration. These results indicated that δ-toxin present on the steady-state skin plays a critical role in the epicutaneous sensitization to food allergen, independently of both mast cells and IL-33-ST2 signaling. However, it is unclear how much of δ-toxin present on skin is enough to induce epidermal sensitization. To solve this question, it will be necessary to quantify δ-toxin in the skin of patients with atopic dermatitis or normal controls that are colonized by *S. aureus* in further experiments.

We speculate that epicutaneously treated δ-toxin present on the non-tape-stripped skin stimulates keratinocytes, the most abundant cell type in the epidermis, to release IL-1α. This local release of IL-1α directly or indirectly stimulates the activation of skin cDC2, which is characterized by the uptake of food allergen by cDC2 in the skin and the translocation of cDC2 from the skin to draining LNs. This, in turn, likely results in skewing of Th2/Tfh with food allergen-specific IgE production. Consequently, intragastric OVA administration in δ-toxin-treated mice can result in food allergic responses that are IgE- and mast cell-dependent. These assumptions are potentiated by several findings. *In vitro* stimulation of murine keratinocytes with δ-toxin induced the release of detectable levels of IL-1α, but not of IL-1β, IL-18, IL-33, or IL-25, although these cell types constitutively released TSLP irrespective of δ-toxin stimulation. Furthermore, δ-toxin increased protein levels of IL-1α in the epicutaneously treated skin in this model. It should be noted that in this model, δ-toxin decreased the percentages of skin cDC2 among total skin cells but increased the percentage of OVA-AF647-engulfed cDC2 among skin cDC2, while δ-toxin increased the numbers of both total cDC2 and OVA-AF647-engulfed cDC2 in axillary LN. In addition, *in vitro* stimulation with IL-1α increased the uptake of OVA-AF647 in MHC Class II^high^ BMDCs. Most importantly, pretreatment with IL-1α Ab abrogated the activation of skin cDC2, OVA-specific IgE production, and OVA-induced food allergic responses in our model. Nonetheless, taking into consideration the important role of TSLP in epicutaneous sensitization (11, 26, 37, 38), it seemed reasonable that IL-1α cooperates with TSLP in the δ-toxin-mediated epicutaneous sensitization to food allergen. However, further examination will be required to completely understand the mechanisms by which locally released IL-1α causes epicutaneous sensitization via cDC2 in this model.

Although δ-toxin is speculated to directly stimulate several types of immune cells through putative δ-toxin receptors, the expression levels of these receptors are extremely low in murine keratinocytes (12, 39). However, δ-toxin exhibited *in vitro* cytotoxicity on murine keratinocytes. Further, stimulation with IL-1α up-regulated mRNA levels of IL-1α in murine keratinocytes. Hence, δ-toxin-induced cell death of keratinocytes likely plays a primary role in the release of IL-1α, which transcriptionally up-regulates IL-1α levels in an autocrine manner.

Recent studies using murine models have reported that in epicutaneous infection of *S. aureus*, PSMα induces the release of IL-1α and IL-36α from keratinocytes, leading to IL-17-dependent skin inflammation (15, 16). Furthermore, IL-36α enhances IgE production by directly acting on B cells (17). Although IL-36α expression in skin is strongly up-regulated in previous studies (15, 17), we did not observe it in δ-toxin-treated skin in our model. It should be noted that we were not able to measure protein levels of IL-36α as specific Ab against IL-36α was commercially unavailable. Given that keratinocytes release protein levels of IL-36α in response to the culture supernatants of *S. aureus* (15), PSMα together with other *S. aureus*-derived factors may increase expression levels of IL-36α. Hence, similar mechanisms may be at play to upregulate IL-36α expression in *S. aureus*-colonized skin of patients with atopic dermatitis.

Interestingly, PSMα3-treated mice exhibited weaker food allergic responses with less frequent diarrhea than δ-toxin-treated mice in our model. In accordance with this, the PSMα3-treated mice exhibited weaker activation of skin cDC2 and lower levels of OVA-specific IgE production than δ-toxin-treated mice before OVA administration. Consistent with the finding that PSMα3 exerts stronger cytotoxicity on murine keratinocytes than δ-toxin, stimulation with PSMα3 more strongly induced the release of DAMPs, including IL-1α, ATP, and HMGB1 than that with δ-toxin. However, δ-toxin more strongly increased the levels of IL-1α, but not of ATP and HMGB1, in skin tissues than PSMα3 in murine model. This may partly explain the different responses between the treatments with PSMα3 and δ-toxin in our study. It is also possible to speculate that PSMα on the non-tape-stripped skin cooperates with specific DAMPs locally released to induce inflammation in a different way from δ-toxin. Accordingly, it seems that δ-toxin present on steady-state skin is prone to skew toward Th2/Tfh with antigen-specific IgE production via keratinocyte-derived IL-1α, although PSMα skews toward Th17 in murine model of epicutaneous *S. aureus* infection (15, 16, 40, 41). In any case, we need to further investigate the mechanisms underlying the different effects of δ-toxin and PSMα on epicutaneous sensitization.

In conclusion, epicutaneous treatment of δ-toxin on non-tape-stripped skin strongly promotes epicutaneous sensitization to food allergens, resulting in food allergy after the uptake of the same allergen. This model may recapitulate epicutaneous sensitization in normal skin colonized by δ-toxin-producing *S. aureus.* Hence, keratinocyte-derived IL-1α plays a critical role in the development of food allergy. Therefore, targeting IL-1α may be an appropriate strategy to prevent the development of food allergy in individuals whose skins are colonized by δ-toxin-producing *S. aureus.*

## Data availability statement

The original contributions presented in the study are included in the article/**Supplementary Material**. Further inquiries can be directed to the corresponding author.

## Ethics statement

All the procedures using mice were approved by the institutional review boards of Juntendo University (approval No. 310050 and 310051).

## Author contributions

HY performed all the experiments and participated in writing the manuscript. AnK, KI, TA, AyK, SU, AM, MK, and RY assisted with the analysis of murine models of food allergy. HW, MN, KM, KU, and NN assisted with the *in vitro* experiments. YO, HO, KO, and TS analyzed the data. JK conceived the project, analyzed the data, and actively participated in manuscript writing. All authors contributed to the article and approved the submitted version.
