# Supplementary material for: Staphylococcus aureus δ-toxin present on skin promotes the development of food allergy in a murine model
Source: Front Immunol. 2023 May 19;14:1173069. doi: 10.3389/fimmu.2023.1173069 (PMC10235538; doi:10.3389/fimmu.2023.1173069)

Supplementary Material

Staphylococcus aureus δ-toxin present on skin promotes the development of food allergy in a murine model

Hiromichi Yamada, Ayako Kaitani, Kumi Izawa, Tomoaki Ando, Anna Kamei[1], Shino Uchida, Akie Maehara, Mayuki Kojima, Risa Yamamoto, Hexing Wang, Masakazu Nagamine, Keiko Maeda, Koichiro Uchida, Nobuhiro Nakano, Yoshikazu Ohtsuka, Hideoki Ogawa, Ko Okumura, Toshiaki Shimizu, Jiro Kitaura*

*** Correspondence:** Corresponding Author: email@uni.edu

Supplementary Table 1. Primer sequences

Gene	primer sequence (forward)	primer sequence (reverse)
mouse *Il1a*	5′-cgcttgagtcggcaaagaaat-3′	5′-tggcagaactgtagtcttcgt-3′
mouse *Il36a*	5′-agagcaaacagttccagtcac-3′	5′-ggcctttgcactcccatgta-3′
mouse *Il4*	5′-ctcatggagctgcagagac-3′	5′-agtgatgtggacttggactc-3′
mouse *Il13*	5′-catggtatggagtgtggacctg-3′	5′-ggtatcggggaggctggaga-3′
mouse *Mcpt1*	5′-gaagctcaccaaggctgca-3′	5′-ccaccaataatctcctcagctcca-3′
mouse *Fpr1*	5′-tccagagctgttggaaagttca-3′	5′-acatccagaacgatgtagcca-3′
mouse *Fpr2*	5′-ccacaggaaccgaagagtgta-3′	5′-accaccacttctgatccattca-3′
mouse *Fpr-rs1*	5′-gccgtcctttccgagttctt-3′	5′-tgcctaaaagggccaccaat-3′
mouse *Fpr-rs3*	5′-tttctgaactccagccgtcc-3′	5′-atgctgcttggtgtctcctt-3′
mouse *Fpr-rs4*	5′-gcaagaggggatgtgtactgt-3′	5′-agctgtggcagcaataacaga-3′
mouse *Fpr-rs6*	5′-tgccatggatcgttgtatttgt-3′	5′-actttcgttgccacattcac-3′
mouse *Fpr-rs7*	5′-gaatctggcaaggaaagtgattttg-3′	5′-cagtgtactttaccactttcatctt-3′
mouse *18S rRNA*	5′-atggtagtcgccgtgcctac-3′	5′-ccggaatcgaaccctgatt-3′

SUPPLEMENTARY FIGURE LEGENDS

Supplementary Figure 1. δ-toxin present on the non-tape-stripped skin upregulated mRNA levels of IL-4 and MCPT-1 in jejunum tissues in murine model of food allergy following epicutaneous sensitization to food allergen. (**A**) Experimental design for food allergy of intragastrically OVA-administered WT mice that had been epicutaneously treated or not with OVA ± δ-toxin once a week for six weeks on the non-tape-stripped abdominal skin. Small intestines were isolated on day 57. (**B-D**) Relative mRNA expression levels of (**B**) IL-4, (**C**) IL-13, and (**D**) MCPT-1 in jejunum tissues from the mice on day 57 after the final administration of OVA. Data are representative of two independent experiments. Means ± SD have been plotted. *$P < 0.05$.

Supplementary Figure 2. Mast cell deficiency did not influence δ-toxin-mediated production of OVA-specific IgE in murine model. (A) Experimental design for epicutaneous sensitization. WT and $Kit^{W\text{-}sh/W\text{-}sh}$ mice were epicutaneously treated with OVA ± δ-toxin once a week for six weeks on the non-tape-stripped abdominal skin. On day 41, blood samples were obtained. (B) Serum levels of OVA-specific IgE in WT and $Kit^{W\text{-}sh/W\text{-}sh}$ mice. Data are representative of two independent experiments. Means ± SD have been plotted. ns, not significant.

Supplementary Figure 3. The deficiency of neither mast cells nor ST2 influenced δ-toxin-mediated increase of AF647-positive cDC2 numbers in axillary LN. (A) Related to Figure 4B, the percentages of skin cDC2 among total skin cells were shown. (B) Related to Figure 4D, total cDC2 in axillary LN. (**C**) Experimental design for analyzing dendritic cells in axillary LN. WT mice were epicutaneously treated with OVA-AF647 + δ-toxin on days 0 and 7 on the non-tape-stripped abdominal skin. Axillary LNs were isolated on Day 8. (**D**, **E**) AF-647-positive cDC2 in axillary LN from (**D**) WT and $Kit^{W\text{-}sh/W\text{-}sh}$ mice and (**E**) WT and ST2 KO mice 24 h after the last treatment. Data are representative of two independent experiments. Means ± SD have been plotted. *$P < 0.05$.

Supplementary Figure 4. In comparison to PSMα3, δ-toxin present on the non-tape-stripped skin induced food allergic responses more strongly following epicutaneous sensitization to food allergen in murine model. (**A**) Experimental design for analyzing dendritic cells in axillary LN. WT mice were epicutaneously treated with OVA-AF647 plus δ-toxin or PSMα3 on days 0 and 7 on the non-tape-stripped abdominal skin. Axillary LNs were isolated on Day 8. (**B**) AF-647-positive cDC2 in axillary LN from the mice 24 h after the last treatment. (**C**) Experimental design for food allergy of intragastrically OVA-challenged WT mice that had been epicutaneously treated with OVA plus δ-toxin or PSMα3 once a week for six weeks on the non-tape-stripped abdominal skin. Blood samples were taken on day 41, and small intestines were isolated on day 57. (**D**) The serum levels of OVA-specific IgE on day 41 before OVA challenges. (**E**) The frequency of diarrhea in OVA-administered mice. (**F**) The numbers of jejunum mast cells of the mice after the final administration of OVA. (**B, D-F**) Data are representative of two independent experiments. Means ± SD have been plotted. *$P < 0.05$ or **$P < 0.01$.

Supplementary Figure 5. mRNA expression levels of putative receptors of δ-toxin were extremely low. (**A**) Relative mRNA expression levels of putative δ-toxin receptors in murine keratinocytes and bone marrow-derived mast cells. (**B-D**) Murine keratinocytes were stimulated with different concentrations of δ-toxin or PSMα3 for 2 h, as indicated. Concentrations of (**B**) IL-1α, (**C**) ATP, and (**D**) HMGB1 in the culture supernatants. (**E**) Murine keratinocytes were stimulated with 50 ng/mL IL-1α for 2 h. Relative mRNA levels of IL-1α. Data are representative of three independent experiments. Means ± SD have been plotted. *$P < 0.05$ or **$P < 0.01$.

Supplementary Figure 6. IL-1α levels in skin tissues were higher in the mice epicutaneously treated with OVA plus δ-toxin than in those with OVA plus PSMα3. (**A**) Experimental design for analyzing the levels of IL-1α, ATP, and HMGB1 in skin tissues. (**B-D**) Levels of (**B**) IL-1α, (**C**) ATP, (**D**) HMGB1 in skin tissue homogenates. Data are representative of two independent experiments. Means ± SD have been plotted. *$P < 0.05$. ns, not significant.

Supplementary Figure 7. Stimulation with IL-1α increased the uptake of OVA-AF647 in MHC Class II^{high} BMDCs. (**A**) BMDCs that had been cultured in the presence of 0 or 10 ng/mL IL-1α for 12 h were incubated with 0, 100, or 300 ng/mL OVA-AF647 for 1 h to measure the percentages of OVA-AF647 among MHC Class II^{high} BMDCs. Data are representative of three independent experiments. Means ± SD have been plotted. **P* < 0.05 or ***P* < 0.01.

Supplementary Figure 8. Pretreatment with anti-IL-1α Ab suppressed the increase in epidermal thickness in murine model of δ-toxin-mediated, OVA-induced food allergy. (**A**) Experimental design for food allergy of intragastrically OVA-administered WT mice that had been epicutaneously treated with OVA + δ-toxin once a week for six weeks on the non-tape-stripped abdominal skin. The effects of intraperitoneal administration of anti-IL-1α Ab or control Ab were examined. Skins were isolated on day 57. (**B**) The thickness of epidermis. (**C**) Skin sections stained with hematoxylin and eosin (scale bars, 100 μm). Data are representative of two independent experiments. Means ± SD have been plotted. ***P* < 0.01.

SUPPLEMENTARY FIGURE 1

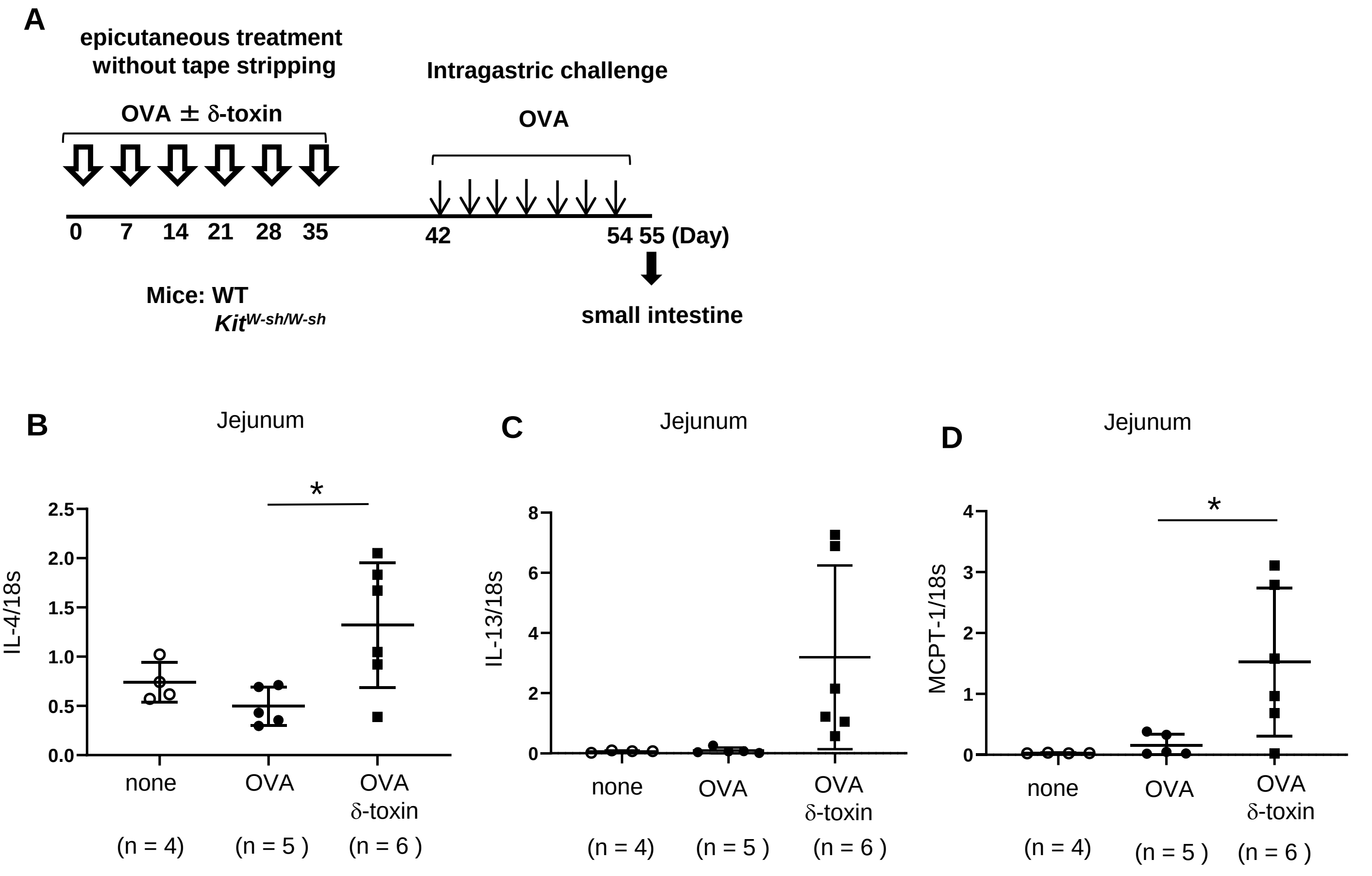

SUPPLEMENTARY FIGURE 2

A

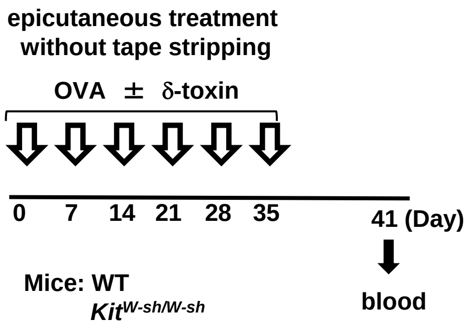

B

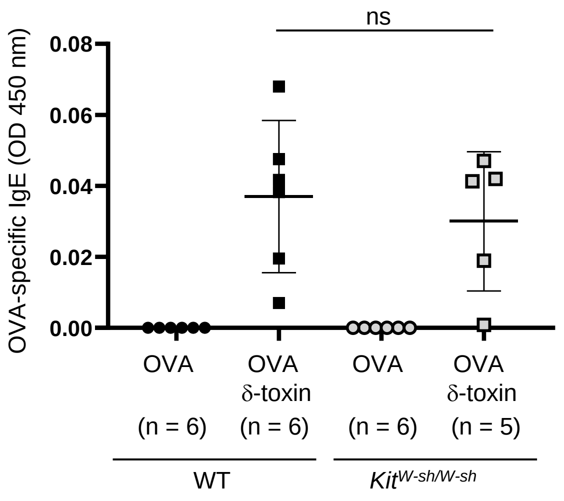

SUPPLEMENTARY FIGURE 3

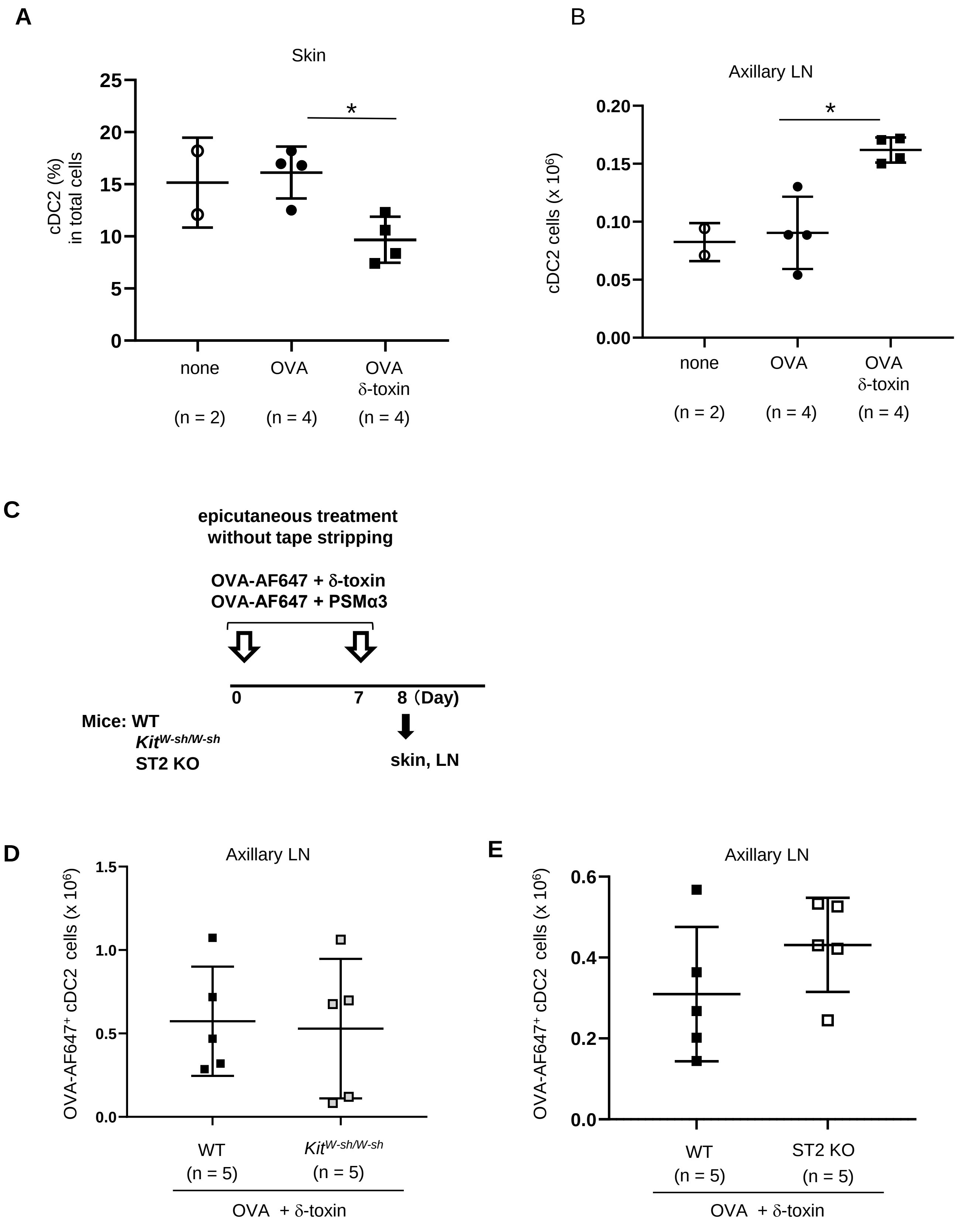

SUPPLEMENTARY FIGURE 4

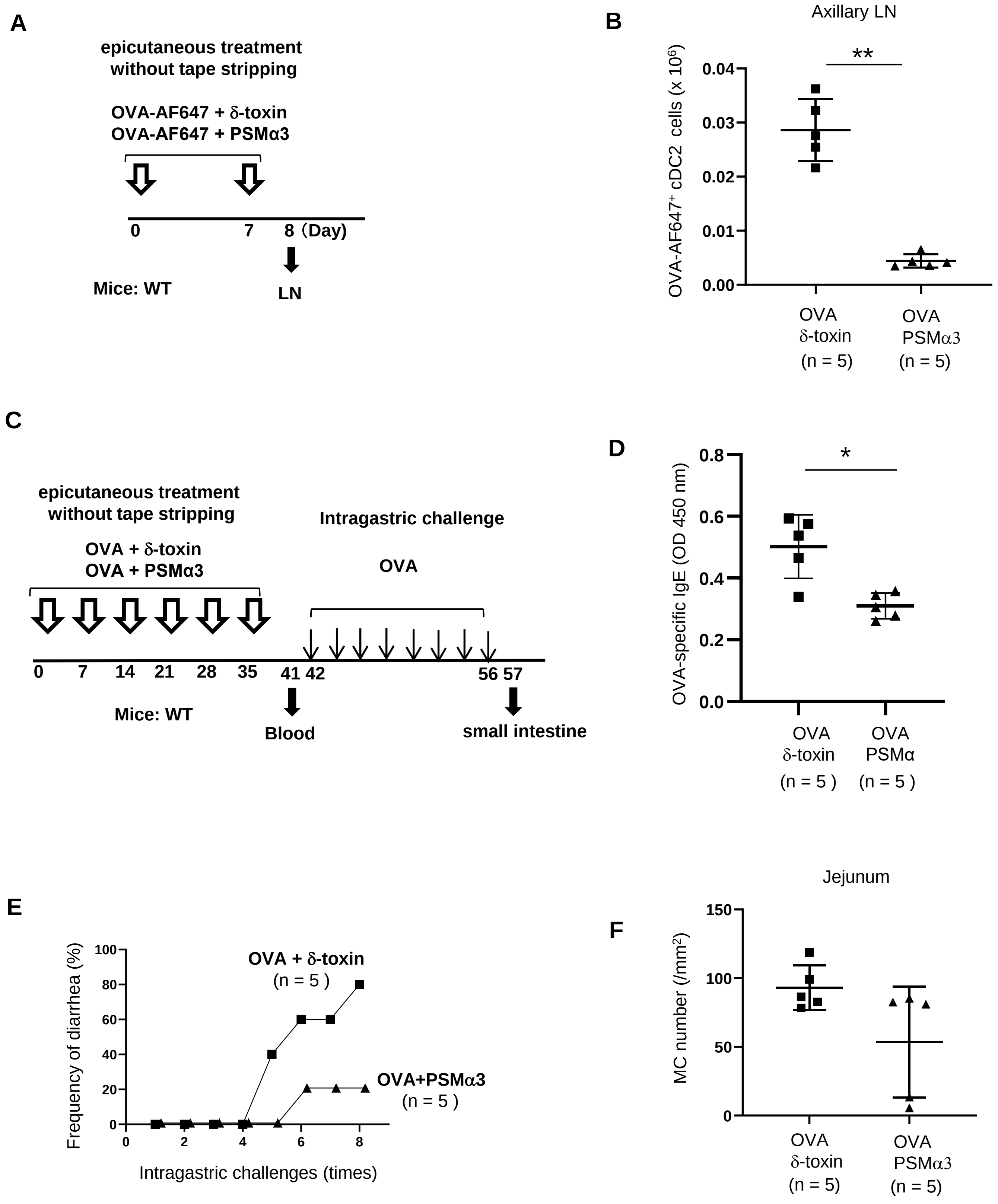

SUPPLEMENTARY FIGURE 5

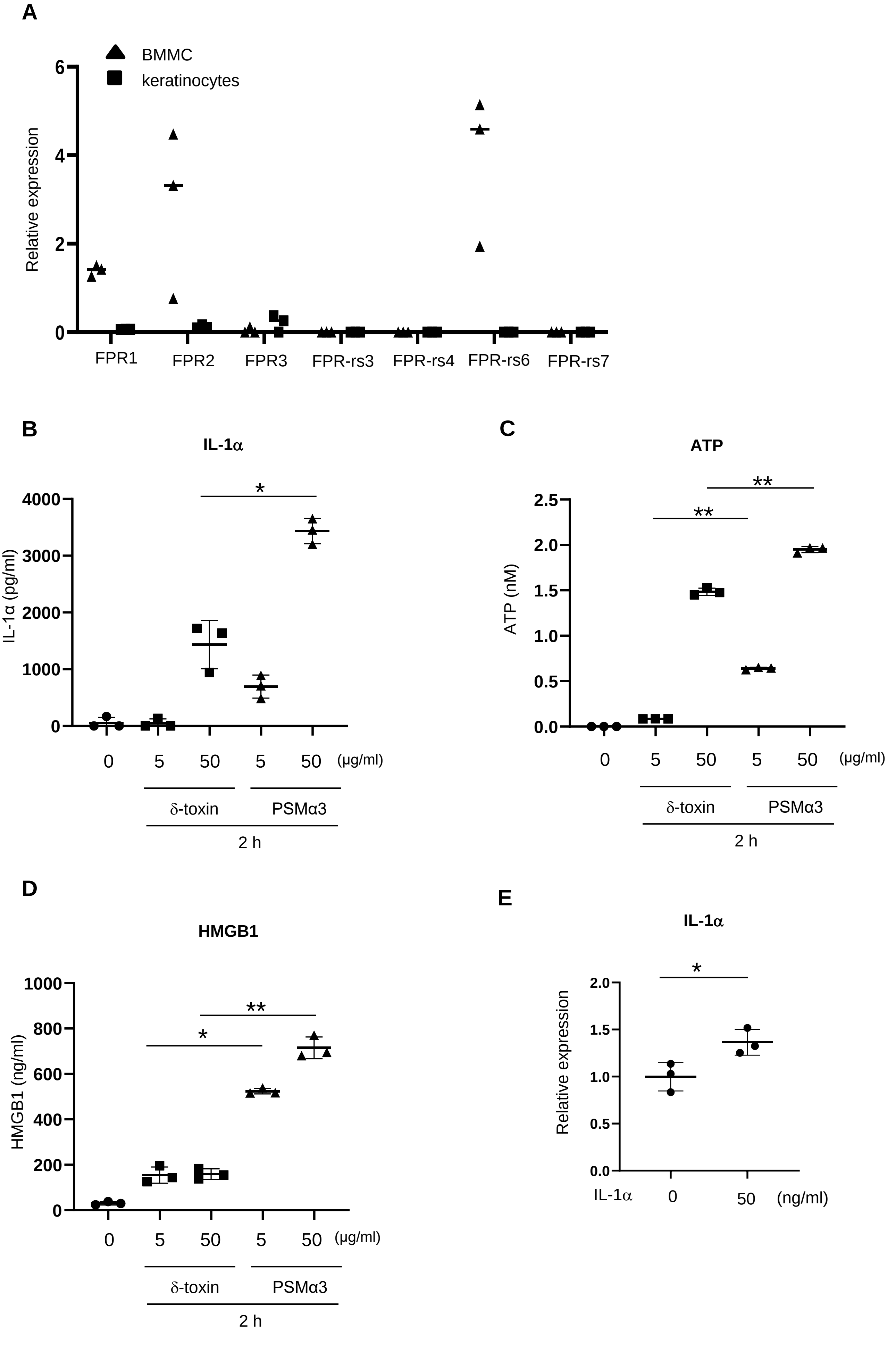

SUPPLEMENTARY FIGURE 6

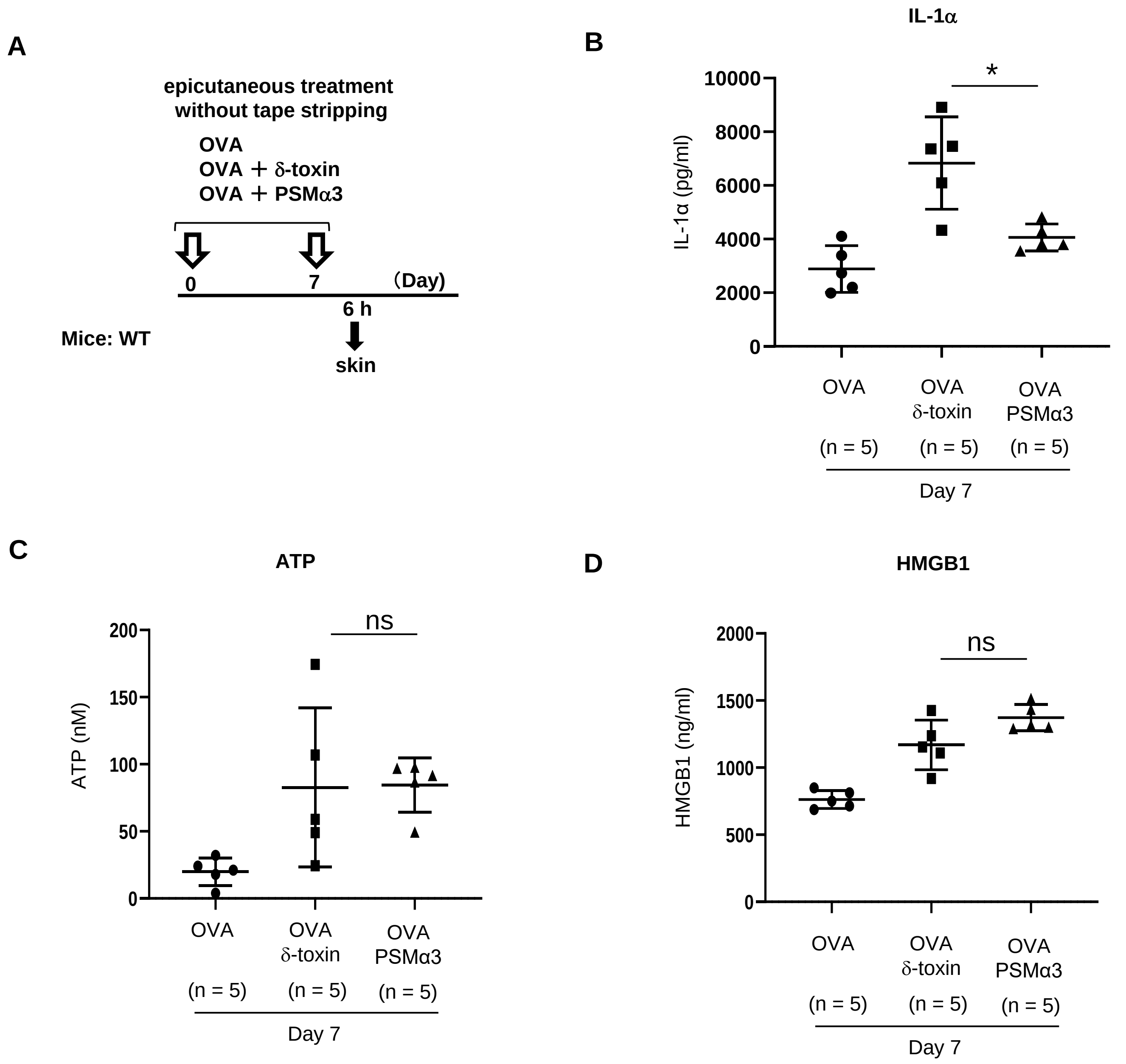

SUPPLEMENTARY FIGURE 7

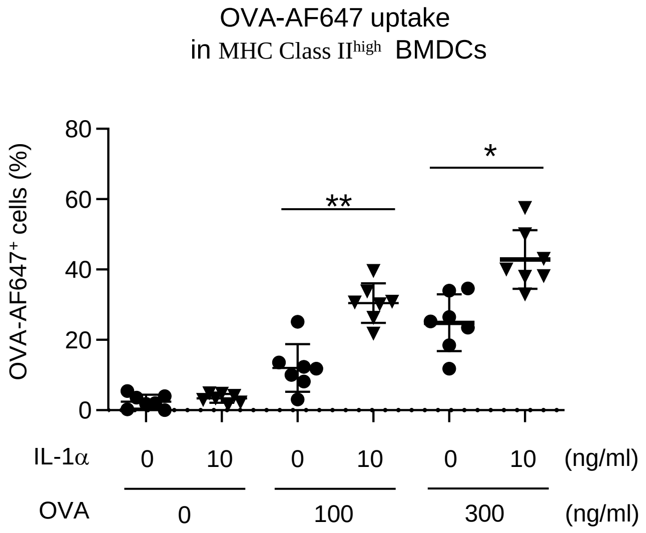

SUPPLEMENTARY FIGURE 8

A

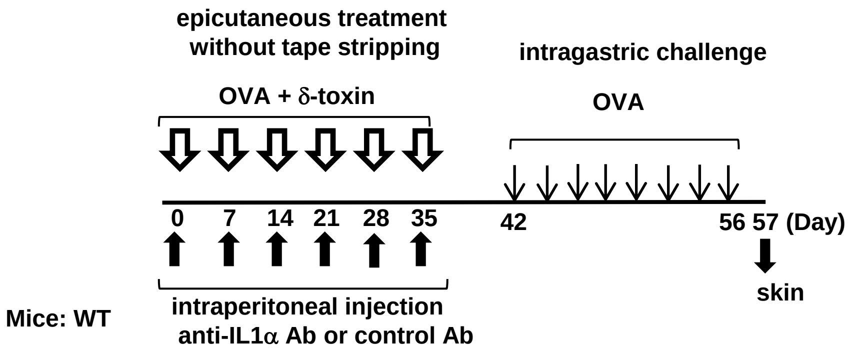

B

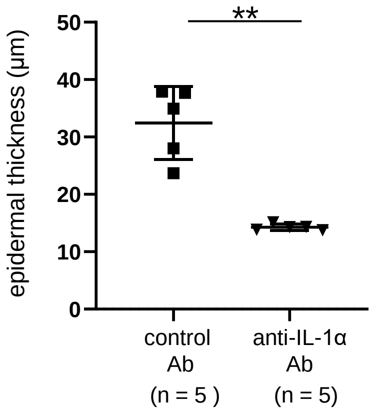

C

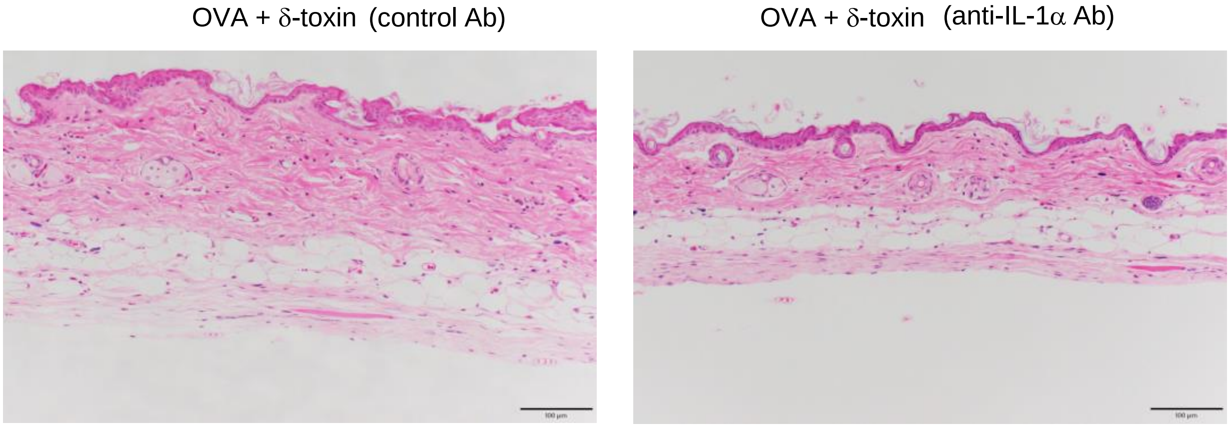

Supplement: Supplementary file 1 [file DataSheet_1.pdf]
